# Predicting Habitat Distribution of Five Heteropteran Pest Species in Iran

**DOI:** 10.1673/031.013.11601

**Published:** 2013-10-26

**Authors:** Samaneh Solhjouy-Fard, Alimorad Sarafrazi, Mehdi Minbashi Moeini, Ali Ahadiyat

**Affiliations:** 1 Department of Entomology, Science and Research Branch, Islamic Azad University, Tehran, Iran; 2 Insect Taxonomy Research Department, Iranian Research Institute of Plant Protection, Evin, Yemen Street, P.O.Box: 19395-1454, Tehran, Iran; 3 Weed Science Research Department, Iranian Research Institute of Plant Protection, Evin, Yemen Street, P.O.Box, 19395-1454, Tehran, Iran

**Keywords:** *Adelphocoris lineolatus*, *Apodiphus amygdali*, distribution models, habitat suitability, *Lygus pratensis*, MaxEnt, *Nezara viridula*, *Nysius cymoides*

## Abstract

In agroecosystems, potential species distribution models are extensively applied in pest management strategies, revealing species ecological requirements and demonstrating relationships between species distribution and predictive variables. The Maximum Entropy model was used to predict the potential distribution of five heteropteran key pests in Iran, namely *Adelphocoris lineolatus* (Goeze) (Hemiptera: Miridae), *Lygus pratensis* (L.), *Apodiphus amygdali* (Germar) (Hemiptera: Pentatomidae), *Nezara viridula* (L.), and *Nysius cymoides* (Spinola) (Hemiptera: Lygaeidae). A total of 663 samples were collected from different parts of Iran. The altitude and climate variable data were included in the analysis. Based on test and training data, the area under the receiver operating characteristic curve values were above 0.80, the binomial omission test with the lowest presence threshold for all species was statistically significant (< 0.01), and the test omission rates were less than 3%. The suitability of areas in Iran for *A. amygdale* (Germar) (Hemiptera: Pentatomidae), *N. cymoides* (Spinola) (Hemiptera: Lygaeidae), *A. lineolatus* (Goeze) (Hemiptera: Miridae), *L. pratensis* (L.), and *N. viridula* (L.) (Hemiptera: Pentatomidae), ranked as 78.86%, 68.78%, 43.29%, 20%, and 15.16%, respectively. In general, central parts of Iran including salt lakes, deserts, and sand dune areas with very high temperatures and windy weather were predicted to be less suitable, while other regions, mainly northern parts, were most suitable. These new data could be applied practically for the design of integrated pest management and crop development programs.

## Introduction

About 60% of Heteroptera, with more than 40,000 described species (Weirauch and Schuh 2011), are phytophagous (Schaefer and Panizzi 2001). The key pests are mainly found in three families, namely Miridae, Pentatomidae, and Lygaeidae. The species *Adelphocoris lineolatus* (Goeze) (Miridae), *Lygus pratensis* (L.), *Apodiphus amygdali* (Germar) (Pentatomidae), *Nezara viridula* (L.), and *Nysius cymoides* (Spinola) (Lygaeidae) are of economic importance in Iran because of their outstanding damage to alfalfa (Yasunaga 1990; Wheeler Jr. 2001; Mirab-balou et al. 2008), cotton (Behdad 2002), clover (Wipfli et al. 1990), canola (Heidary Alizadeh et al. 2009; Sarafrazi et al. 2009), pistachio nuts (Mehrnejad 2001), almond, and apple (Ghauri 1977). Despite using chemical and biological control measures against these insect pests, their damage is still outstanding, which could partly be related to the lack of information on some biological aspects and the impact of different climates on their performance.

The modeling established based on environmental variables and occurrence species records helps growers in their pest management strategies. The modeling could be used in management of invasive insect species in new areas (Roura-Pascual et al. 2009; Solhjouy Fard 2011; Wilson et al. 2011), determining the effect of global climate change on pest (Franklin 2009) and their host plant distributions (Ulrichs and Hopper 2008), revealing species ecological requirements relationships between the distribution of species and predictive variables as well as the importance of each variable in model building (Araújo and Guisan 2006).

Many models with different statistical algorithms, such as maximum entropy modeling (MaxEnt), genetic algorithm for rule-set prediction (GARP), DOMAIN, BIOCLIM, limiting variable and environmental suitability (LIVES), and so on (Elith et al. 2006; Phillips et al. 2006), predict species potential distribution with presence-only data. MaxEnt, as a good choice for presence-only data (Elith and Leathwick 2009), has demonstrated a high predictive accuracy (Elith et al. 2006). This method predicts based on Maximum Entropy (i.e., closest to the uniform) (Phillips et al. 2006). It requires presence-only data, randomly selected background data from the study area as pseudo-absence, and environmental variables to generate predictions (Phillips et al. 2006). It indicates probability of species occurrence in each grid cell of the study region (Stachura-Skierczynska et al. 2009) based on environmental conditions where the species have been observed.

This is the first study on distribution modeling and predicting the geographic distribution of some heteropteran pest species in Iran. It was conducted to complete a part of their zoogeographical information that could be used in a thorough designing of management strategies based mainly on ecological niche modeling.

## Materials and Methods

### Species records

**Figure 1.**
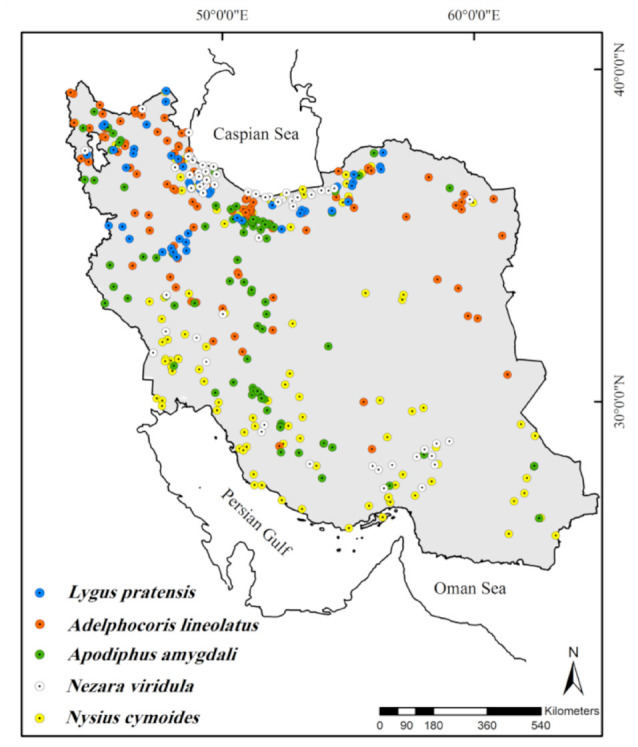
Current distribution map of five heteropteran pest species in Iran. High quality figures are available online.

In order to determine the distribution model of *Ad. lineolatus*, *L. pratensis*, *Ap. amygdali*, *Ne. viridula*, and *Ny. cymoides*, a total of 663 samples, *(Ad. lineolatus* 189, *L. pratensis* 69, *Ap. amygdali* 105, *Ne. viridula* 81, *Ny. cymoides* 219), including those collected from different parts of Iran, records of the species deposited in the Hayk Mirzayans Insect Museum in the Insect Taxonomy Research Department, and species published in related documents (Hoberlandt 1954, 1995; Linnavuori 2007a, 2007b, 2008) (Figure 1) were used in the niche ecological modeling. The geographical coordinates for some localities were obtained and/or corrected using Google Earth (www.googleearth.com). The data was checked in ArcGIS software (ESRI 2008) for any errors.

### Environmental variables

Environmental variables are generally selected based on species ecology (Holway et al. 2002; Roura-Pascula et al. 2009). The variables, including annual mean temperature, mean diurnal range, maximum temperature of warmest month, minimum temperature of coldest month, annual precipitation, and precipitation of the wettest and driest months, were downloaded from the WorldClim dataset (Hijmans et al. 2005). These particular climate dimensions were chosen to represent environmental dimensions relevant to distribution and survival of small arthropods (De Meyer et al. 2010). The selected data were in raster format with 30 arc second (∼1 km) spatial resolutions (Hijmans et al. 2005).

### Model building and evaluations

Because of its better performance than the other species distribution models (Elith et al. 2006), The MaxEnt software (version 3.3.3e) was utilized for modeling. Models were calibrated using 75% of the available records for each species as training data, and the remaining 25% were used for model validation as test data. The model settings were 10000 randomly selected background points as pseudoabsence in the entire studied area, regularization multiplier 1, and 1000 maximum iteration with 10^-5^ convergence threshold. Logistic output format was used to describe the probability of presence (Phillips and Dudık 2008), which is a continuous habitat suitability range between 0 (unsuitable) and 1 (the most suitable). MaxEnt was run ten times for each species in order to get average prediction. The outputs in ASCII format were proprocessed and visualized using ArcGIS 9.3® (ESRI 2008). The Jackknife analysis was used to indicate the most informative variables.

The accuracy and performance of species distribution models were evaluated using threshold-independent receiver operation characteristic (ROC) analysis (Elith et al. 2006; Phillips et al. 2006) and thresholddependent binomial test of omission (Phillips et al. 2006). The area under the ROC curve (AUC) ranges between 0 and 1. Models with an AUC value higher than 0.75 are acceptable models (Pearce and Ferrier 2000). In the second test, the lowest presence threshold (Pearson et al. 2007) was selected to represent the areas that were at least as suitable as those where the species has been recorded (Hernandez et al. 2006; Pearson et al. 2007). Omission rates in optimal models are less than 0.05 (Anderson et al. 2003).

## Results

**Figure 2.**
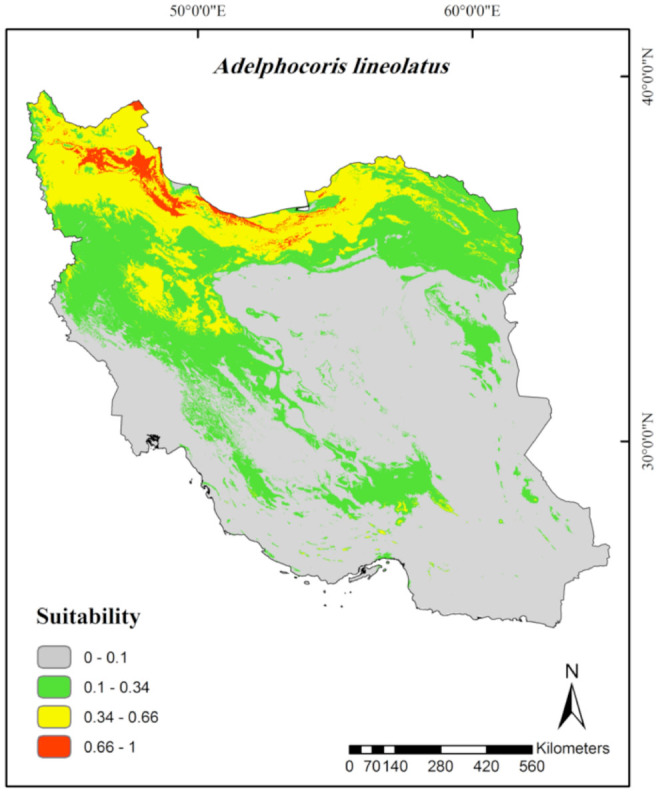
Prediction of habitat suitability for *Adelphocoris lineolatus* in Iran. High quality figures are available online.

**Figure 3.**
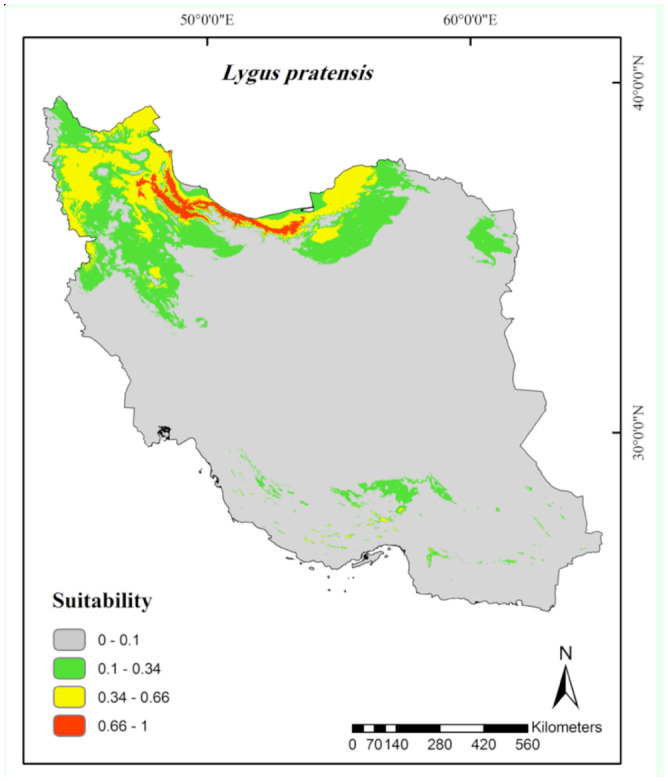
Prediction of habitat suitability for *Lygus pratensis* in Iran. High quality figures are available online.

**Figure 4.**
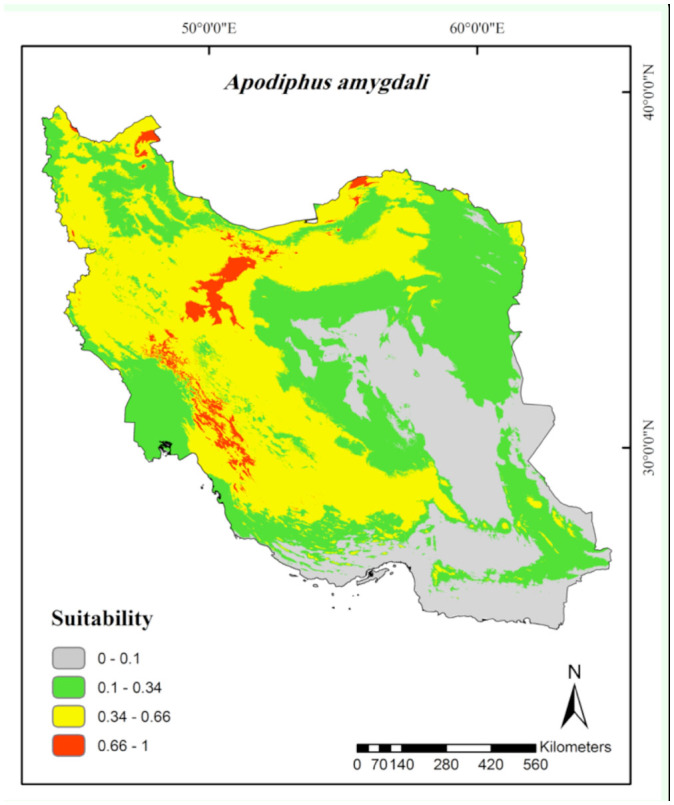
Prediction of habitat suitability for *Apodiphus amygdali* in Iran. High quality figures are available online.

**Figure 5.**
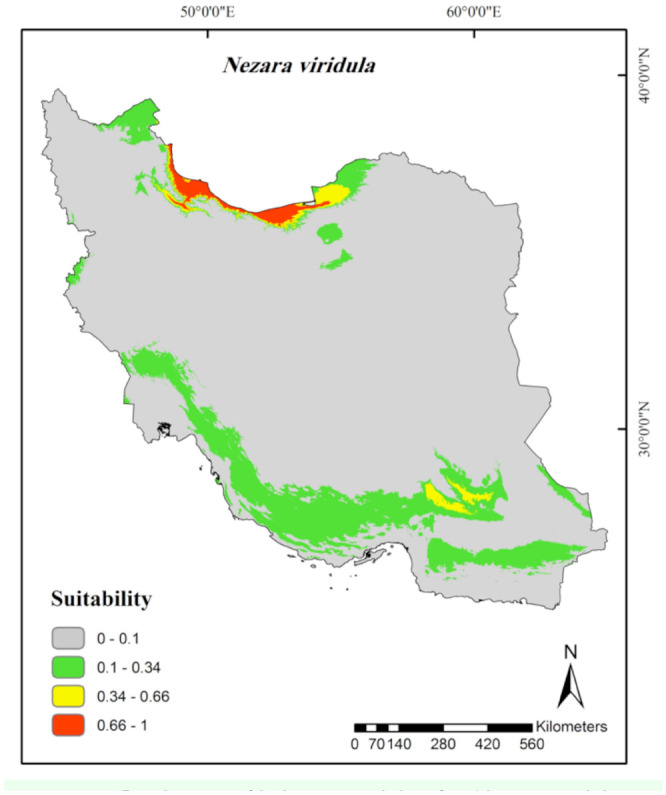
Prediction of habitat suitability for *Nezara viridula* in Iran. High quality figures are available online.

**Figure 6.**
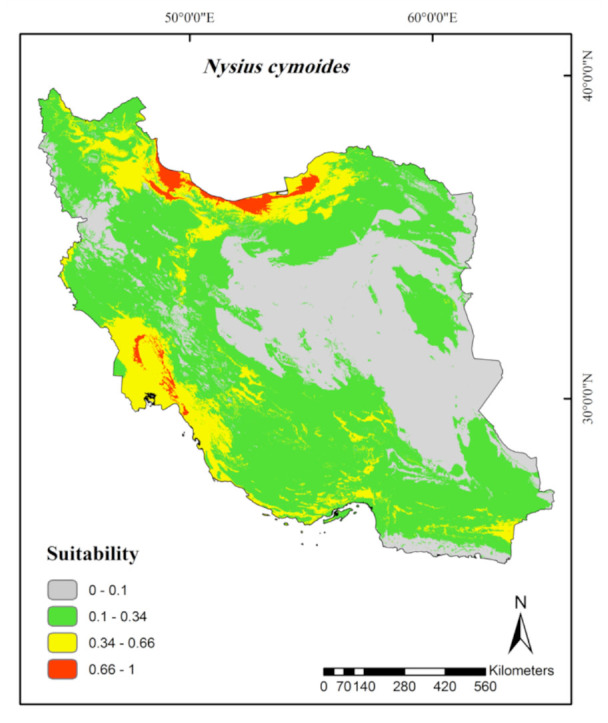
Prediction of habitat suitability for *Nysius cymoides* in Iran. High quality figures are available online.

According to the continuous average maps (Figures 2 to 6), 43.29% of the studied area, mainly northern parts of Iran, was considered as a suitable habitat for *Ad. lineolatus*. The suitable areas for *L. pratensis* and *Ne. viridula* were also extended to some parts of southern Iran, comprising 20% and 15.16% of the studied area respectively. Most parts of Iran (78.86%), except deserts, were considered as suitable habitats for *Ap. amygdali*. The geographic distribution map predicted *Ny. cymoides* to occur in most of the areas (68.78%), except central and eastern Iran. In general, the central part of Iran was predicted as the least suitable area, and the northern part was predicted as the most suitable.

**Table 1.**
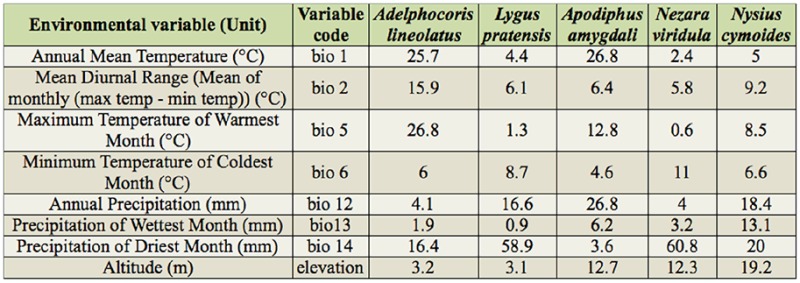
The percent contribution of environmental variables in predicting the species geographic distribution models. For description of each variable, see text.

**Figure 7.**
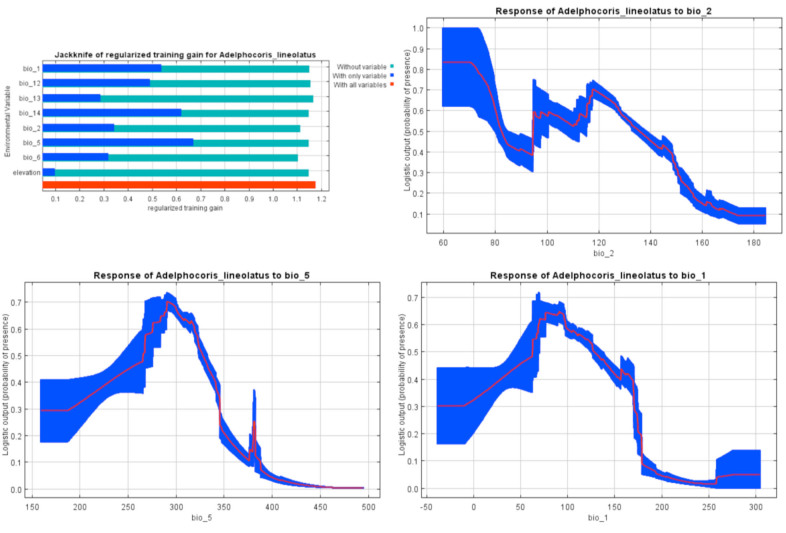
MaxEnt Jackknife tests of the environmental variable importance and marginal response curves of the predicted probability of *Adelphocoris lineolatus* occurrence for explanatory variables that contributed substantially to the Iran MaxEnt model. High quality figures are available online.

The Jackknife test of variable importance showed that maximum temperature of warmest month (26.8%), annual mean temperature (25.7%), precipitation of driest month (16.4%), and mean diurnal range (15.9%) were the most important factors in *Ad. lineolatus* habitat distribution prediction (Table 1). According to the marginal response curves, the probability of occurrence was maximal when maximum temperature of warmest month and annual mean temperature ranged between 25–32° C and 5–20°C respectively. The precipitation of the driest month should also range between 5–35 mm. A negative relationship between probability of occurrence and mean diurnal range was observed (Figure 7).

**Figure 8.**
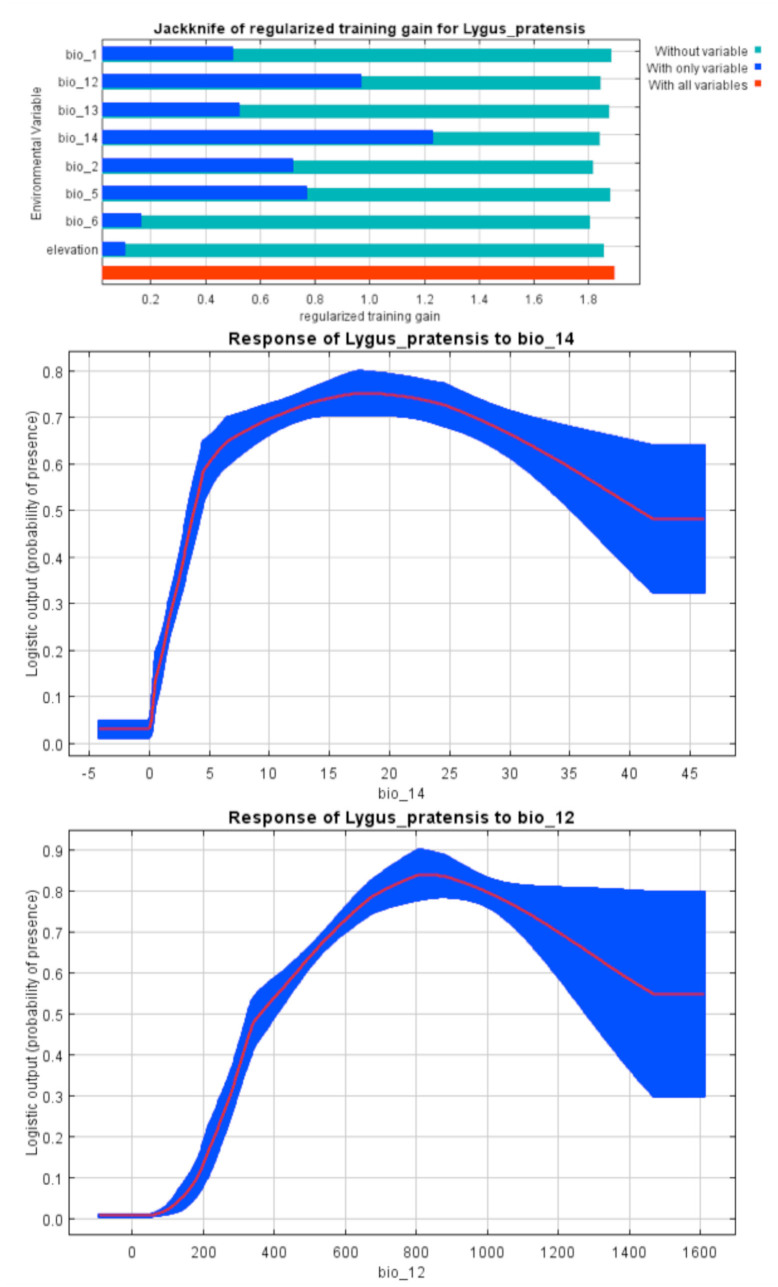
MaxEnt Jackknife tests of the environmental variable importance and marginal response curves of the contribution variables to predict presence probability of *Lygus pratensis* with MaxEnt in Iran. See Table 1 for definition of bio variables. High quality figures are available online.

Among the eight variables, precipitation of driest month (58.9%) and annual precipitation (16.6%) had the highest contribution for *L. pratensis* modeling construction (Table 1). The species occurrence showed an increase when the amount of precipitation during the driest months and annually was less than 20 and 800 mm respectively. The percentage was then decreased with increasing precipitation (Figure 8).

**Figure 9.**
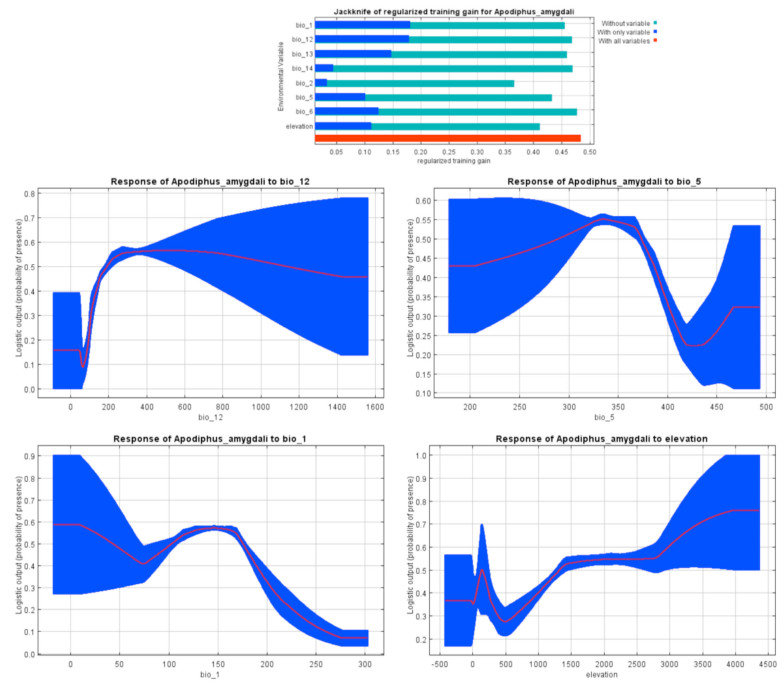
MaxEnt Jackknife tests of the environmental variable importance and marginal response curves of the contribution variables to predict presence probability of *Apodiphus amygdali* with MaxEnt in Iran. See Table 1 for definition of bio variables. High quality figures are available online.

The highest contribution in prediction modeling of *Ap. amygdali* belonged to annual precipitation (26.8%), annual mean temperature (26.8%), maximum temperature of warmest month (12.8%), and altitude (12.7%) (Table 1). Marginal response curves showed positive relationships between maximum temperature of warmest month and altitude, and a negative association between annual mean temperature and the species occurrence (Figure 9). The highest probabilities of occurrence were in areas with less than 37 mm annual precipitation.

**Figure 10.**
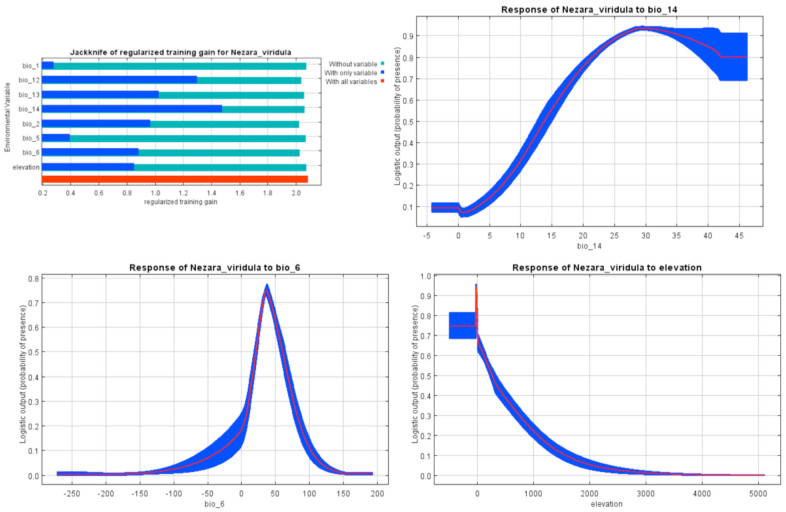
MaxEnt Jackknife tests of the environmental variable importance and marginal response curves of the contribution variables to predict presence probability of *Nezara viridula* with MaxEnt in Iran. See Table 1 for definition of bio variables. High quality figures are available online.

Precipitation of driest month (60.8%), altitude (12.3%), and minimum temperature of coldest month (11%) had the greatest contribution in determining of *Ne. viridula* habitats in Iran (Table 1). The occurrence probability of *Ne. viridula* indicated a positive relationship with precipitation of driest month, while the peak of occurrence was between 0–7° C minimum temperatures of coldest month. Higher altitudes were predicted to be suitable habitats (Figure 10).

**Figure 11.**
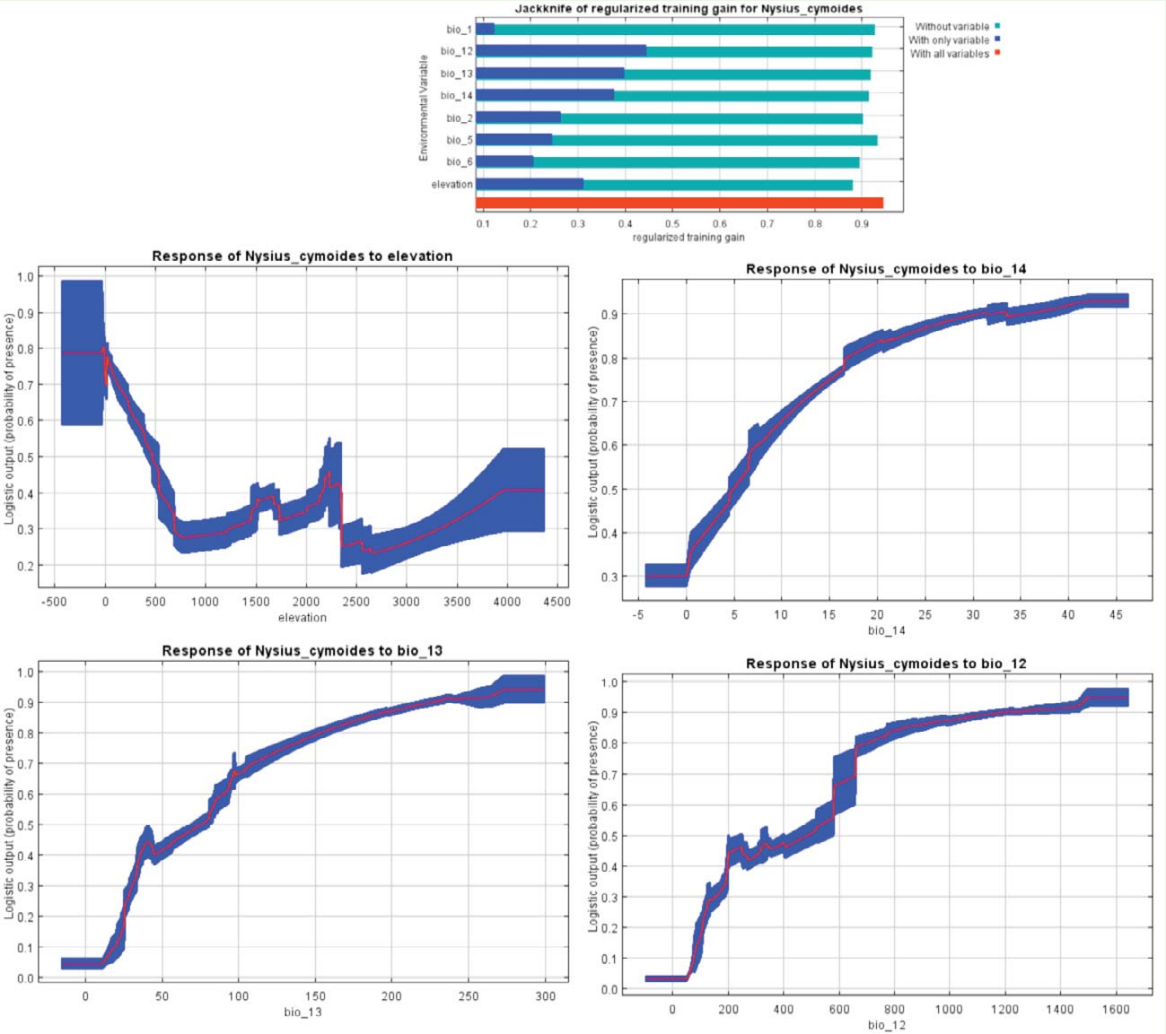
MaxEnt Jackknife tests of the environmental variable importance and marginal response curves of the contribution variables to predict presence probability of *Nysius cymoides* with MaxEnt in Iran. See Table 1 for definition of bio variables. High quality figures are available online.

For *Ny. cymoides* modeling, the relevant environmental variables were precipitation of driest (20%) and wettest month (13.1%), altitude (19.2%), and annual precipitation (18.4%) (Table 1). A positive relationship was observed between precipitation of driest month, annual precipitation, and precipitation of wettest month and the probability of the species occurrence, but this relationship was negative in terms of altitude (Figure 11).

MaxEnt results generated two ROC curves, displaying AUC values, for each species based on training and test data. The AUC values based on training and test data were respectively 0.92 and 0.87 (SD = 0.02) for *Ad. lineolatus*, 0.96 and 0.94 (SD = 0.01) for *L. pratensis*, 0.85 and 0.80 (SD = 0.03) for *Ap. amygdali*, 0.94 and 0.92 (SD = 0.02) for *Ne. viridula*, and 0.88 and 0.84 (SD = 0.02) for *Ny. cymoides* (Table 2). Therefore, the performances of the models were good for predicting habitat suitability in training and test locations. The binomial omission test with the lowest presence threshold for all species was statistically significant, and the test omission rates were very low, not exceeding 2% (Table 2).

**Table 2.**
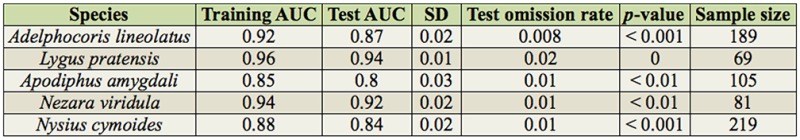
Statistical evaluation of MaxEnt model using threshold-dependent and threshold-independent evaluations. The *p-*value obtained using the minimum training presence

## Discussion

According to the obtained models, besides the Iran climate classifications (de Pauw et al. 2002), the semi-humid, humid, and posthumid parts of the Irano-Turanian zoogeographical subregion, which has host plants such as alfalfa and clover (Ministry of Agriculture 2010), were identified as suitable areas for *Ad. lineolatus* distribution. The pest is also found in Mediterranean subregions (including many parts of Europe and Mediterranean regions of North Africa) (Kerzhner and Josifov 1999), Siberia, Central Asia, North America, and Canada (Wheeler Jr. 2001; Grichanov and Ovsyannikova 2009), areas with humid and cold to temperate climates. The marginal response curves (Figure 7) showed that the probability of occurrence has a direct relationship with the amount of precipitation. Some small regions within the southern parts of Iran (Figure 2) and dry areas, except regions with high temperatures (> 30° C) (de Pauw et al. 2002), were moderately to poorly suitable for the pest. Marginal response curves showed a decrease of occurrence probability in higher temperatures. The results implied that *Ad. lineolatus* prefers humid environments with low temperatures, while the altitude does not significantly affect its distribution. The lack of the pest occurrence record in Saharo-Arabian zoogeographical subregion (Konstantinov et al. 2009), including the southern part of Iran, Iraq, Saudi Arabia, and parts of northern Africa (Kerzhner and Josifov 1999), could partly be related to the high temperatures of the localities. Central parts of Iran including salt lakes, deserts, and sand dune areas with high temperatures and windy weather (Bakhtiyari 1998) were unsuitable habitats for the species, probably because of the impact of hot, dry, and windy weather on the insect embryo development (Grichanov and Ovsyannikova 2009).

The most suitable areas for *L. pratensis* (Figure 3) occurred in humid and semi-humid regions of Khouzestan and Fars provinces (Ministry of Agriculture 2010), especially in areas with warm summers (10–30° C), where its host plants, alfalfa and canola, are extensively planted. The distribution model predicted some parts of southern Iran, where no records are available, as suitable areas for the pest. At least two reasons, including the incomplete sampling and/or biotic factors such as interspecific competitions, could be the reason for this prediction. The suitable ar eas for *L. pratensis* in Iran, such as the coast of the Caspian Sea (Gilan Province), are mostly similar to those of Mediterranean climate (Badripour 2004) where the species has already been recorded (Kerzhner and Josifov 1999). According to the marginal response curves (Figure 8), a positive relationship between precipitation and species occurrence was observed. More humid (> 20 mm for driest months and > 800 mm for annual precipitation) and post-humid areas were less suitable. Dry regions had low to moderate suitability for the pest. Climate regions such as the central part of Iran, with arid to hyperarid climates, including salt lakes, deserts, and sand dune areas (Badripour 2004), with high temperature (> 30° C) were unsuitable habitats. Humid regions and areas with warm summers (10–30° C) are generally preferred habitats for adults. The marginal response curves showed that the species has mostly been distributed in areas with lower temperatures. *Lygus* bugs are able to tolerate habitats with low temperatures, such as canola growing regions in Canada and Sweden (Otani and Carcamo 2011).

*Ap. amygdali* is a key pest of pistachio nuts (Mehrnejad 2001), almond, and apple (Ghauri 1977) in Iran. Most parts of the studied areas, except hyper-arid and arid regions (e.g., the central plateau and southeast of Iran, which have coastal dry climates and very warm summers (> 30° C)), planted with the abovementioned plants (Ministry of Agriculture 2010) were predicted as suitable habitats (Figure 4). The pest has been recorded throughout Palaearctic regions (Rider 1999). According to the Jackknife test and marginal response curves (Figure 9), the probability of the species occurrence decreases in areas with very high temperatures, lower precipitations, and lower altitudes. Based on Javahery's (1994) study, low relative humidity (< 20%) limits development and emergence of several pentatomid species. The pest has been collected from areas with altitudes of 41 to 3500 m a.s.l. (mainly above 1500 m a.s.l.). A report by Ghauri (1977) showed that the *Apodiphus* species were found in lower hills at altitudes up to 1600 m a.s.l. rather than in higher regions of Iran, Turkey, and countries further west. This contradiction could mostly be related to Ghauri having only a few sampling sites.

In terms of *Ne. viridula*, China (1938) stated that the pest could be considered as a cosmopolitan species, except for in the colder regions. The species is spread throughout the Palaearctic region, especially in tropical and subtropical parts (Rider 1999). According to the Iran climate classification (de Pauw et al. 2002) and response curves, semi-arid to humid regions with higher precipitation and lower altitudes (Tougou et al. 2009) are positively associated with *Ne. viridula* (L.) occurrence. The temperature response curves and the results of Clarke and Walter (1993) implied that the species could survive in low temperatures. Furthermore, the increasing of aridity reduces the chance of the species occurrence. Under laboratory conditions, the nymphs move to areas with higher humidity (Hirose et al. 2006). This means that the emergence from eggs and the survival of the nymphs are strongly dependent on high relative humidity. Localities with these kinds of characteristics are mostly found in costal parts of the Caspian Sea with low altitudes and southern provinces, including Bushehr, Hormozgan, and southern parts of Kerman and Sistan-Balouchestan (Figure 5) as a part of the Saharo-Arabian zoogeographical subregion. The main growing areas for the two main host plants of *Ne. viridula*, cotton and pistachio, are Golestan in the north and the southern provinces of Iran, respectively (Ministry of Agriculture 2010).

According to the habitat distribution model (Figure 6), *Ny. cymoides* showed mostly the same results as *Ap. amygdali*. The pest has been recorded from most fields, semi-desert areas, and steppic areas of the Palaearctic region (Pericart 1999; Linnavuori 2007b). On the contrary, deserts, salt lakes, sand dune areas (Badripour 2004), and coastal arid regions (e.g., coast of Oman Sea) (Bakhtiyari 1998) are considered as unsuitable habitats. According to Iran climate classifications, the presence records, and the marginal response curves, *Ny. cymoides* prefers humid regions. Regardless of an exceptional record of the pest at an altitude of 2660 m a.s.l., the results of Jackknife test and response curve indicate the negative impact of altitude on the species distribution (Figure 11). This is probably why the insect prefers low lands such as the coast of the Caspian Sea and the Khouzestan plains. The regions are cultivated with its main host plants, such as Canola (Ministry of Agriculture 2010).

In congruence with several already published studies (e.g. Elith et al. 2006; Crawford and Hoagland 2010; Wilson et al. 2011), the MaxEnt showed its high ability to produce prediction distribution models for the species under study.

References to the AUC values, omission of test points, and the significant predictions of the results opened new insights into the ecology and climatic-based distribution of the pests. These outstanding new data could be applied practically in designing integrated pest management and crop development programs.

## Abbreviations

AUC: area under receiver operating characteristic curve
MaxEnt: maximum entropy modeling
ROC: receiver operating characteristic
